# Absolute Structure from Scanning Electron Microscopy

**DOI:** 10.1038/s41598-020-59854-y

**Published:** 2020-03-04

**Authors:** Ulrich Burkhardt, Horst Borrmann, Philip Moll, Marcus Schmidt, Yuri Grin, Aimo Winkelmann

**Affiliations:** 1 Max-Planck-Institut für Chemische Physik fester Stoffe, Dresden, Germany; 2 École polytechnique fédéral de Lausanne, CH-1015 Lausanne, Switzerland; 3 Laser Zentrum Hannover e.V, Hannover, Germany; 4 Present Address: Academic Centre for Materials and Nanotechnology, AGH University of Science and Technology, 30-059 Krakow, Poland

**Keywords:** Surfaces, interfaces and thin films, Structure of solids and liquids

## Abstract

The absence of centrosymmetry in chiral and polar crystal structures is the reason for many technical relevant physical properties like optical birefringence or ferroelectricity. Other chirality related properties that are actually intensively investigated are unconventional superconductivity or unusual magnetic ordering like skyrmions in materials with B20 structure. Despite the often close crystal structure - property relation, its detection is often challenging due to superposition of domains with different absolute structure e.g. chirality. Our investigations of high quality CoSi crystals with B20 structure by both complementary methods *X*- ray (volume sensitive) and electron backscatter diffraction (EBSD) (surface sensitive) results the consistent assignment of the chirality and reveal fundamental differences in their sensitivity to chirality. The analysis of the surface of a CoSi crystal with domains of different chirality show the high spatial resolution of this method which opens the possibility to analyze the chirality in microstructures of technical relevant materials like thin films and catalysts.

## Introduction

Non-centrosymmetry is a fascinating and challenging topic and has great relevance in many fields of chemical and physical research, material science and crystallography. In case of crystalline materials, both enantiomorphs of a non-centrosymmetric phase crystallize in different absolute structures with complementary chirality^1–3^ or polarity. Absence of centrosymmetry is a necessary condition of various physical and chemical properties which are subject of highly active research areas such as unusually ordered magnetic^4,5^ and superconducting states^6,7^, topological surface properties^8,9^ or enantioselective catalysis on non-centrosymmetric materials^10–13^. Respective experiments crucially depend on efficient methods to reliably identify the chirality, enantiomorphic purity, and crystallographic orientation of crystals and crystallites. Especially, the spatial resolution on microscopic scales relevant for modern technological applications is of special interest in this field due to possible non-trivial effects of the microstructure on macroscopic properties^14^. In this respect, the scanning electron microscope (SEM) offers considerable practical advantages, as it is a high-throughput, analysis instrument, and manufacturing tool, which in principle can cover large samples with resolution down to the nanometer scale. In order to demonstrate the potential of SEM measurements for fast microscopic determination of the chirality, we apply dynamical electron diffraction in the SEM to determine the local chirality in the sample, and we verify the results by absolute structure determination using X-ray diffraction methods. This combination results in a powerful new tool for spatially resolved analysis of the chirality in crystalline materials.

Among the common experimental methods which are sensitive to non-centrosymmetry of a crystal structure, X-ray and electron diffraction are most often used for intermetallic phases. By exploiting the effects of anomalous scattering^15^, absolute structure determination is routinely carried out using *X*-ray diffraction^16,17^. Compared to *X*-ray investigations in the single-scattering limit, electron diffraction methods exhibit strong dynamical scattering effects, which are sensitive to the absence of centrosymmetry. In this way, chirality can be proven by transmission electron microscopy (TEM) down to the atomic scale^18–22^. Another important effect in the TEM is related to electron channeling, where localized sources of secondary element-specific *X*-rays can reveal the absence of a inversion symmetry center in a crystal structure^23^. Compared to TEM and *X*-ray diffraction, however, the scanning electron microscope has considerable practical advantages. On one hand, sample preparation is often much less demanding than for TEM, while on the other hand, spatial resolution of an SEM is much higher than that of conventional *X*-ray diffraction. Arguably the most important electron diffraction effect in the SEM is the diffraction of incoherently backscattered electrons (backscattered Kikuchi diffraction, BKD), which is applied for determination of crystallographic orientation and phase discrimination in the commonly used techniques of electron backscatter diffraction (EBSD)^24^. With continuing progress in the understanding of Kikuchi diffraction physics, it has been shown that SEM Kikuchi patterns carry essential information about non-centrosymmetric, polar (achiral) and chiral crystal structures beyond the Laue symmetry of the investigated crystal structures^25–28^. It has been shown that chemical etch pits indicating the polar direction in GaP (space group *F* $\bar{4}$3 m) agree with the assignment of the polar direction based on EBSD investigations^29^. Whilst the basic possibility of discriminating of both enantiomorphs of α-quartz (space groups *P*3_1_21 and *P*3_2_21) has been demonstrated via Kikuchi diffraction in the SEM^27^, we present the spatially resolved mapping of chirality and crystallographic orientation of two enantiomorphs that show the symmetry of the same space group. Consequently, the crystal structure is chiral but space group is not – or to state differently the Euclidean normalizer of the space group is centrosymmetric^2,30^. This implies possible ambiguities in the distinction of enantiomorphs and motivates the presented experimental realization. It has to be stated that - not only in the presented analyses – the comparison of experimentally determined chirality of a crystal structure demands the use of right-handed sets of axes at every step of the experimental setup and calculations^16^. Of particular danger are basis transformations of a unit cell into standard setting.

**Figure 1:**
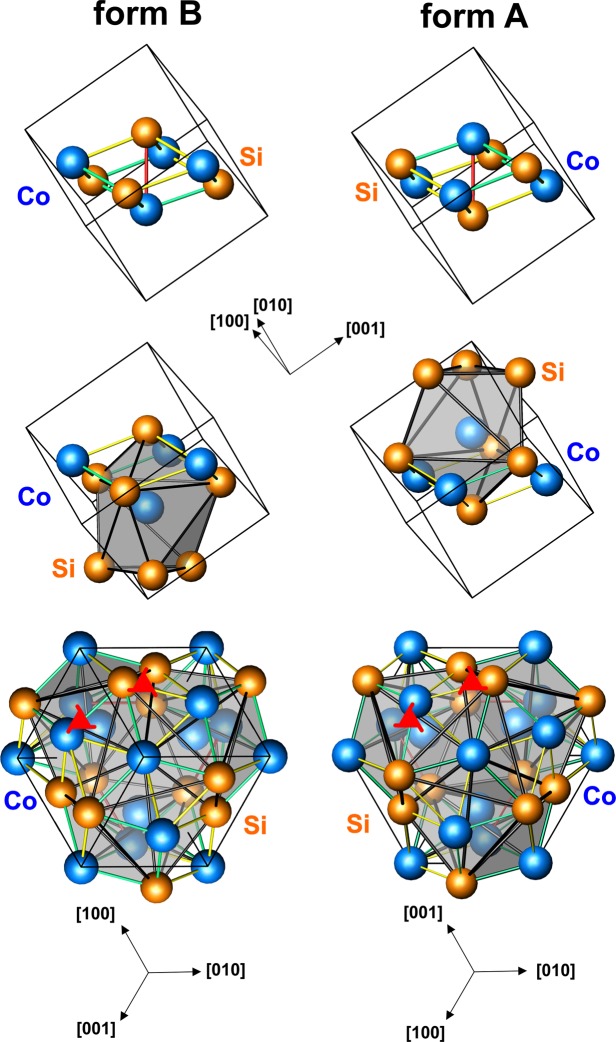
Both enantiomorphic forms (**A** form and **B** form, notation after^31^) of the crystal structure of CoSi (structure type FeSi, Pearson symbol *cP*8): (top) eight atoms of the unit cell with the shortest interatomic distances of 2.31 Å (red), 2.34 Å (yellow), 2.45 Å (green); (middle) coordination polyhedron around the cobalt atoms. Unit cell orientation are identical for drawings in top and middle row; (bottom) view along the three-fold axis visualizing the different chirality of both absolute structures; the screw axes 3_1_ and 3_2_ (red triangles) are arranged parallel to the threefold axis.

For our investigation, we have chosen single crystals of CoSi with the cubic FeSi type (B20) structure (space group *P*2_1_3). The unit cell contains eight atoms and is related to the centrosymmetric sodium chloride structure type (space group ${Fm}\bar{3}{m}$). In FeSi type structure, both elements occupy position 4*a* (*x*, *x*, *x*) with *x* deviating from the coordinates ¼ and ¾, that are characteristic for the positions in the NaCl structure type. The asymmetric shift (*x*_Co_ + *x*_Si_ ≠ 1) of the atomic positions along the space diagonal leads in CoSi to a coordination number change from CN = 6 (NaCl) to CN = 7. The regular coordination octahedron in NaCl is distorted by maintaining trigonal symmetry and one face is additionally capped by an atom on the 3-fold axis (Fig. 1, middle). There are three types of shortest interatomic distances (Fig. 1, top), instead of the uniform spacing in the regular octahedron in NaCl. This structure type neither contains an inversion center nor any mirror symmetry. Consequently, the crystal structure is chiral but the handedness is hard to visualize. In fact, the crystal structure and its enantiomorphic counterpart show the symmetry of same space group *P*2_1_3. i.e. left-handed and right-handed screw axes - 3_1_ and 3_2_ - are part of the symmetry of both absolute structures (Fig. 1 bottom). In contrast to chiral structures with screw axis of one hand only-like left-handed and right-handed α-quartz - the orientation of a screw axis is not suitable to assign the chirality of the CoSi crystal structure. The chirality of enantiomorphs of the structure type FeSi was labeled for the first time by Spence *et al*.^31^. According to this notation, the A form shows the atomic positions *x*_Fe_ = 0.1358 and *x*_Si_ = 0.844 for Fe and Si, respectively. The B form defines the inverse. We use this notation for CoSi throughout this paper.

In the following, we will assign the absolute structure of CoSi single crystals by comparison of experimentally measured Kikuchi patterns with advanced Kikuchi diffraction pattern simulations^32^ for both enantiomorphs. In a second step, the absolute structures of the same crystals are confirmed from *X*-ray diffraction data. The main goal of our investigation is to demonstrate the use of EBSD Kikuchi pattern for an SEM–based discrimination of enantiomorphs which have the symmetry of the same space group. Additionally, the concordant, independent assignment of the chirality by EBSD and *X*-ray diffraction proves the consistent use of right-handed set of axes of experimental setups and calculations of both methods. As an application example which underline the advantages of the EBSD analysis, we present spatially resolved EBSD mapping to show the built-up of a CoSi crystallite by domains with different absolute structure.

### Crystal structure determination by *X*-ray diffraction

**Figure 2:**
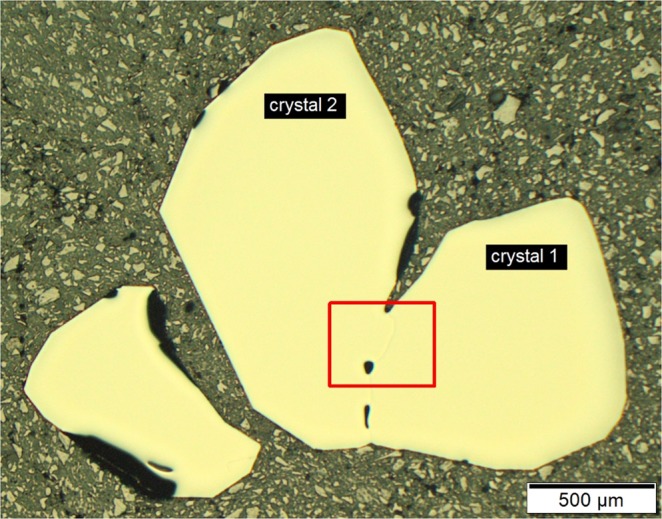
Metallographic cross-section of agglomerated CoSi crystals (optical micrograph; bright field); Crystals are prepared by chemical vapor transport; red frame defines the interface area shown in Fig. 3.

**Figure 3:**
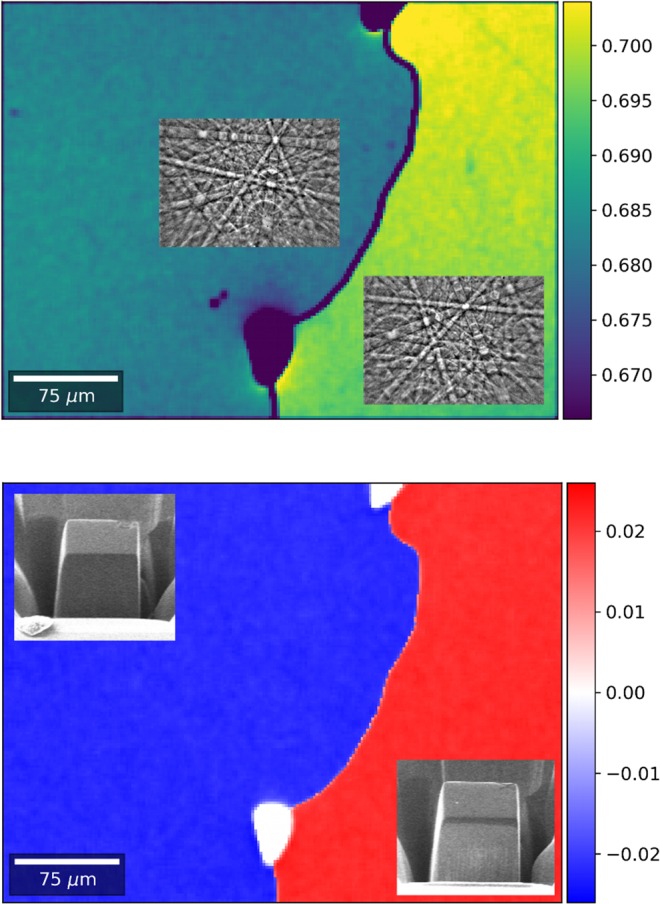
Crystallographic characterization of two as grown CoSi crystallites with different absolute structures: (top) mean value *r*_m_ = ½ (*r*_+E_ + *r*_−E_) of cross correlation coefficients *r*_+E_, *r*_−E_ that measure the coincidence between images of measured EBSD pattern and simulated pattern of both enantiomorphs (inset: experimental Kikuchi pattern); (bottom) difference Δ*r* = (*r*_+E_ – *r*_−E_) of cross correlation coefficient. The sign of Δ*r* is connected to the absolute structure due to the better coincidence between its simulated pattern and the experimental pattern ((+)…A-form; (−)…B-form); insets: FIB specimens used for *X*-ray diffraction measurements, before liftout.

Two cubes with almost the same dimensions were cut by focused ion beam (FIB) technique from crystallite 1 and 2 of the cross-section shown in Fig. 2. The cubes are shown in the inset of Fig. 3 (bottom part) before lift-out. The structure determinations reveal that both crystals represent different absolute structures of the FeSi type. According to the given notation, crystal 1 and 2 show A and B form, respectively (Table 1). The Flack parameters are zero within 1–2 esds (estimated standard deviation) and indicate the enantiomorphic purity of both crystals. Both crystals show a very high quality and also comparable mosacity and absorption effects result in high quality data sets and lead to small reliability values – for example *R*_F_ values are close to 1%. Obviously, quality of the crystals was not significantly affected by the FIB cutting procedure. The results reveal that the coordinates in both enantiomorphs due to anomalous scattering effects are not exactly the same and mutual exchange of Co and Si is accompanied with small shifts along the space diagonal of the cubic unit cell. Detailed analysis of Bijvoet pair intensity differences shows the expected change in sign with almost the same magnitude when changing the absolute structure. For distinct Bijvoet pairs deviation from this general behavior exist. Characteristically affected are e.g. Bijvoet pairs (111)/($\bar{1}11$) with Δ*F*^2^ = −74 and +215 as well as (124)/($\bar{1}24$) with Δ*F*^2^ = +34 and −50 for crystals 1 and 2, respectively (Supplementary Table S1).

**Table 1:** Crystallographic information for the CoSi crystals 1 and 2.

	Crystal 1 (A form)	Crystal 2 (B form)
Composition	CoSi	
Space group	*P*2_1_3	
Pearson symbol	*cP*8	
Temperature	293 K	
Lattice parameter *a*, Å	4.4470(2)	4.4478(2)
Number of reflections for lattice parameter refinement	1361	1362
Number of reflections measured	1755	1784
2*θ*_max_; max sinθ × *λ*^−1^	91.01°; 1.006	90.99°; 1.005
Number of reflections unique	252	251
Measured range	−8 ≤ *h* ≤ 8	−5 ≤ *h* ≤ 6
	−8 ≤ *k* ≤ 8	−8 ≤ *k* ≤ 8
	−6 ≤ *l* ≤ 7	−8 ≤ *l* ≤ 8
*R*_int_	0.040	0.045
Number of reflections with │*F*│ > 4σ*F*	250	250
Extinction coefficient	1.12(3)	1.22(4)
Number of refined parameters	7	7
Flack parameter^17^	−0.014(15)	−0.033(17)
Goodness-of-fit	1.03	1.03
*R*_F_; *wR*_F2_	0.0123; 0.0165	0.0133; 0.0208
*x*_Co_	0.14354(3)	0.85651(4)
*x*_Si_	0.84360(7)	0.15645(8)
ADP_Co_: *U*_11_, *U*_12_*	0.00434(5); 0.00008(4)	0.00439(7); 0.00006(4)
ADP_Si_: *U*_11_, *U*_12_*	0.00461(8); −0.00048(9)	0.00482(9); −0.0004(1)

*Symmetry constrains: U_11_ = U_22_ = U_33_; U_12_ = U_13_ = U_23_.

### Assignment of chirality by EBSD analysis

The EBSD investigations were performed on the CoSi crystallites shown in Fig. 2. Within the cutting plane both crystallites 1 and 2 form a grain boundary with small cavities. The common interface is indicated by crystal morphology and a tiny relief due to metallographic finishing. Both crystallites show different crystallographic orientation (s. EBSD pattern in inset Fig. 3 top) and standard EBSD analysis revealed no further orientation variation within each crystal. Single crystal quality was confirmed by an additional, perpendicular cut at the grain boundary. The standard evaluation of EBSD patterns offered by commercial EBSD systems is not sensitive to non-centrosymmetry. The main reason is that the intensity differences of Friedel pairs are not recovered in pattern simulation models based on kinematic electron scattering model. Also, the signals of weak Kikuchi bands are neglected. Our pattern simulations account for dynamical electron scattering, and comparison with experimental EBSD patterns is realized by a full pattern matching approach. The comparison of complete patterns allows to include the information of weak Kikuchi bands as well as the intricate intensity variations along and in between the bands. To quantify the image similarity between the experimental and simulated data in the pattern matching approach, the normalized cross-correlation coefficient *r* (0 ≤ *r* ≤ 1) is calculated. To discriminate the effect of non-centrosymmetry the cross-correlation coefficient *r*_+E_ and *r*_−E_ for both enantiomorphs are calculated. For crystallite 1 and 2, the mean values *r*_m_ = ½(*r*_+E_ + *r*_−E_) are in the range *r*_m_ = 0.67…0.70 (Fig. 3 top). These values indicate an overall very good agreement between experiment and simulation. The dark areas correspond to cavities at the crystallite interface. The slightly different levels of *r*_m_ for both crystals indicate that the size of the correlation coefficient in the pattern matching is marginally biased by the crystal orientation, i.e. it is influenced by the actual intensity distribution of the specific Kikuchi bands and zone axes which are visible in the Kikuchi pattern. The assignment of the absolute structure is based on the different quantitative match between the experimental and simulated patterns of both absolute structures. In crystallite 1, the difference between the two cross correlation coefficients (Δ*r* = *r*_+E_ − *r*_−E_) amounts Δ*r* =  +0.02 and in crystal 2 the difference is Δ*r* = −0.02. This is visualized in Fig. 3 (bottom). The different sign of ∆*r* in both regions indicates that both crystallites show different absolute structure. In crystal 1, the matching of the experimental with simulated pattern of the A form is better, and in crystal 2, the B form results in a better match. A conservative estimate based on a statistical error analysis indicates, that for a reliable assignment of the absolute structure, changes of |∆*r*| ≥ 0.01 for *r* - values of *r* ≥ 0.5 can be considered to be larger than would be expected from noise or an experimental bias (Supplementary, estimation of the significance of Δ*r*). This estimate also includes the influence of the so-called “excess-deficiency effect”^33^ which describes the apparent illumination of Kikuchi bands from the top in the experimental pattern. The observed values of |∆*r*| for both enantiomorphs are in line with previous estimations for pseudo-symmetry effects in Kikuchi patterns from non-centrosymmetric materials^26^. The specific assignment of the absolute structure to crystallits 1 and 2 agrees completely with the results of the *X*-ray diffraction experiments revealing the full consensus of both characterization methods.

**Figure 4:**
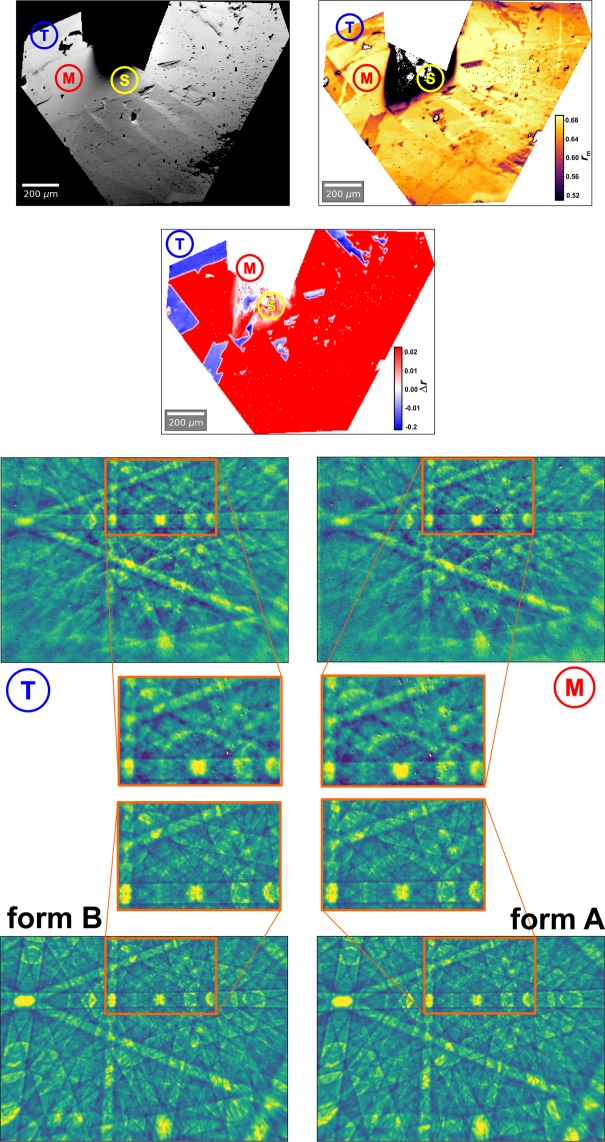
Surface of a CoSi crystallite showing domains of different chirality; (top left) SEM image (SE contrast; 70° tilt corrected; s.a. Supplementary Figure S1); (top right) mean value ${r}_{m}=\frac{({r}_{+E}+{r}_{-E})}{2}$; of cross co*r*relation coefficients *r*_+*E*_,*r*_−*E*_ that quantify the coincidence between images of measured EBSD pattern and simulated pattern of both enantiomorphs, (second row) cross correlation coefficient difference ($\Delta r={r}_{+E}-{r}_{-E}$), red regions result from better coincidence with EBSD pattern of the A form, blue regions result from better coincidence with EBSD pattern of the B form of the CoSi crystal structure, (middle) measured EBSD patterns from region “T” and “M” with magnified details below, (bottom) simulated EBSD patterns of the **A** and **B** form of the CoSi crystal structure with enlarged details above. The detail images (orange frames) visualize the high coincidence between measured and simulated pattern as well as chirality dependent differences in intensity and shape of local spots (see for example upper left corner with localized and broadened spots).

In combination with the calibration of the absolute structure that was achieved by the *X*-ray characterization, the excellent spatial resolution provided by the EBSD method allows to characterize the local chirality of multi-domain crystals. Figure 4 shows EBSD mapping and chirality assignment of domains of a CoSi crystallite. This sample was prepared by chemical vapor transport process as well. The EBSD measurements are performed on the as-grown surface of the crystal without further metallographic preparation. The backscattered electron (BSE) image of the sample is shown in Fig. 4 (top left) (Supplementary, Fig. S1: light optical image). The EBSD measurements were carried out in a polygonal masked part of the total map area of 400 × 300 points at a step size of 3.8 µm. The region marked by ‘S’ is shadowed by another crystal outside of the masked area (Fig. 4 top). The experimental Kikuchi pattern shown in Fig. 4 (middle) are measured in the regions ‘M’ and ‘T’ with a resolution of 320 × 228 pixels. The slowly varying background part in the raw Kikuchi patterns has been removed by high-pass filtering to emphasize the diffraction signal. In order to allow a direct comparison with simulated Kikuchi patterns of the A form and B form (Fig. 4 bottom), all patterns have been normalized to zero mean and unit standard deviation. The values of the average cross correlation *r*_m_ = 0.64–0.68 (Fig. 4 top right) reflect the high quality of the experimental pattern and the very good agreement with the simulations in almost the whole masked area with exception of area ‘S’ with values *r*_m_ <0.52 due to shadowing. Values of the cross correlation differences are in the range ∆*r* = +/−0.02. The mapping of ∆*r* in Fig. 4 (top, second row) visualize that the main part is characterized by ∆*r* ≈ +0.02 e.g. the A form is realized in these areas. In smaller regions with ∆*r* ≈ −0.02 the inverted crystal structure is present. In most of the masked area these ∆*r* values are sufficiently high to distinguish between both absolute structures. In the shadowed area ‘S’ the small values r_m_ does not allow a reliable assignment of the absolute structure by the cross correlation values.

**Figure 5:**
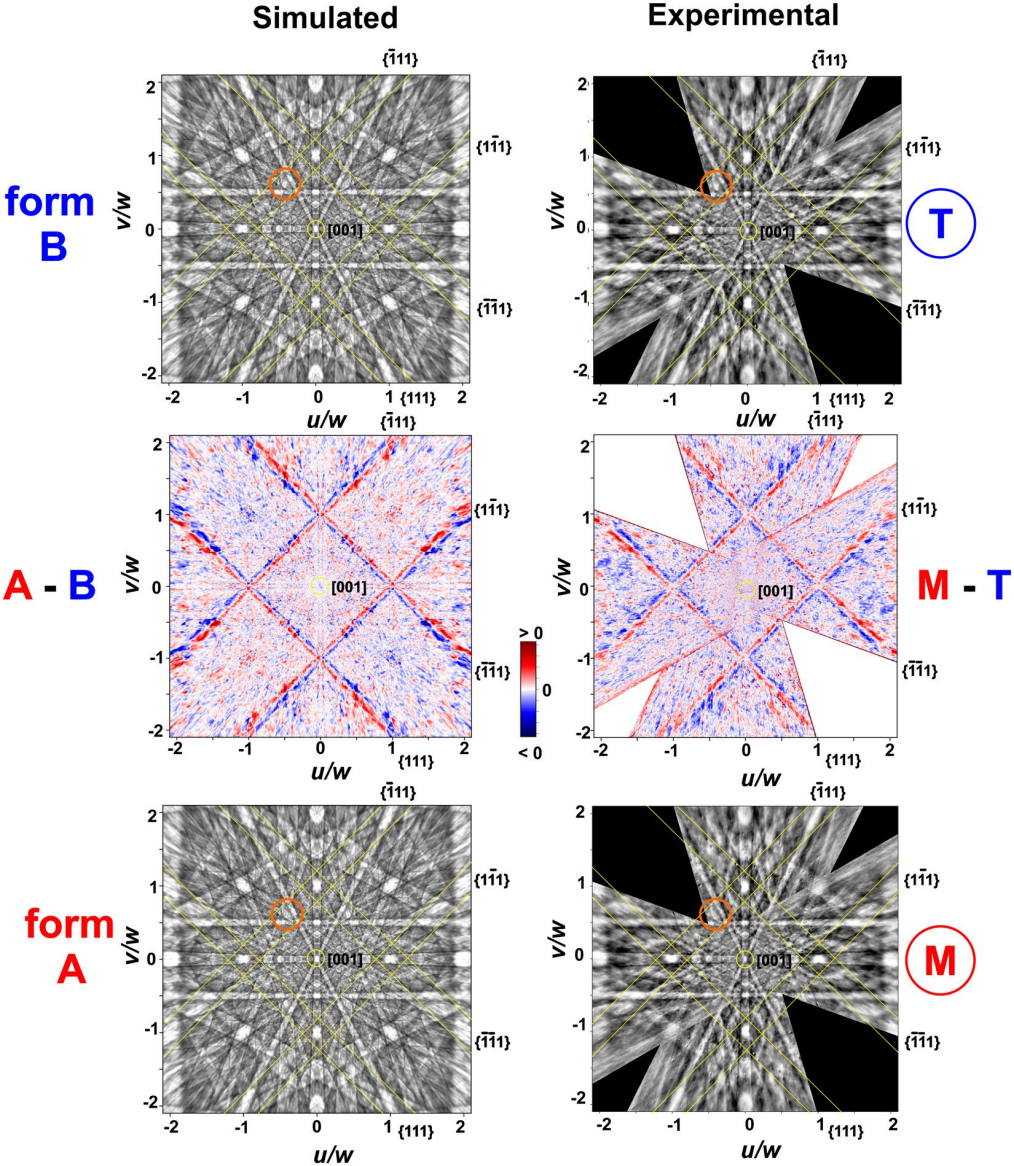
Simulated (left, top and bottom) and measured (right, top and bottom) EBSD pattern transformed by a gnomonic projection to the crystal coordinate system. The [001] zone axis at the center. The indices (*hk*1) of the Kikuchi bands are given by their intersection with the *h* – and *k* - axes. The orientations of the {111} bands are shown by yellow margins. The orange circle is related to the orange frames in Fig. 4. The A–B (middle left) and “M”–“T” visualize the differences in the simulated and the measured EBSD patterns, respectively. Most significant are parallel red and blue lines along the {111} Kikuchi bands. They represent the chirality dependent shift of intensity in the {111} bands.

The Kikuchi pattern from regions ‘M’ and ‘T’ (Fig. 4 middle) as well as the simulated pattern of the A and B form (Fig. 4 bottom) look very similar due to the very close crystallographic orientation. This allows to focus on the small but significant differences in the EBSD pattern that originate from the chirality of the investigated crystal structure. On one hand, intensity and shape of local spots changes if the measuring point moves from the “T” to the “M” region as shown in the magnified details in Fig. 4 middle. These differences are found in a very similar way between the simulated pattern of the A and B form (Fig. 4 bottom, magnified details). On the other hands, the intensity distribution in the {111} Kikuchi bands change significantly as shown in Fig. 5 (s.a. Supplementary Figure S4, alternate pattern presentation). In Fig. 5, the patterns are transformed by a gnomonic projection to the crystal coordinate system. Whilst the [001] zone axis are located at the center, the indices of the Kikuchi bands (*hk*1) are given by their intersection with the *h* and *k* axes, here. For example, the {$\bar{1}11$} bands intersect the *h*-axis at *h* = −1 under 45°. In the simulated pattern of the B form (Fig. 5 top left) the intensity maximum of this centerline. In the pattern of the A form (Fig. 5 bottom left) the maximum intensity is below the centerline of the {$\bar{1}11$} band. The intensity shift affects all {111} lines in the same way. This is visualized in the difference image A – B (Fig. 5 middle left). In the white regions both patterns are identical. The colored, parallel lines show the chirality dependent shift of intensity in the {111} Kikuchi bands. A very similar behavior is observed in the measured EBSD pattern of the “T” and “M” region (Fig. 5 right column;). The very good agreement between both difference images results that the A form of the CoSi structure is present in the “M” region and “T” region shows the invers chirality of the B form. This is conform to the assignment of cross correlation method (Fig. 5 top second row).

The {111} Kikuchi bands are actually rather inconspicuous in the EBSD pattern of the CoSi phase (Fig. 5 top and bottom; yellow margins). It can be difficult to recognize their critical role if a comparison to pattern from a region with different chirality and equal crystallographic orientation is not available. Because of the key role of the {111} bands, ∆*r* depends on the location of these bands in the measured Kikuchi pattern. As can be seen from Fig. 5, the {111} bands cross in the <101> and <011> zone axes, which are 45° away from the <001> directions. In a cubic lattice, the solid angle captured by the EBSD setup can always be adjusted by a change in the camera length to safely contain {111} bands, independent of the actual orientation of the crystal (for example, the vertical and horizontal viewing angles are 63° and 91° for the patterns seen in Fig. 4. The concentration on the effect in the {111} bands also suggests to separate the problems of establishing the local Laue group resolved crystal orientation and detecting the influence of the non-centrosymmetric effects. Instead of analyzing the full Kikuchi patterns with small effects on average, one could restrict the enantiomorph test only to the most relevant Kikuchi bands. The (*hkl*) indices of the corresponding lattice planes (in our case {111}) can be selected beforehand from pattern simulations like in Figs. 4 and 5, while the locations of the relevant Kikuchi bands in the experimental pattern are determined by the local Laue group orientation without considering chirality at all. In this way, the assignment of the absolute structure can be focused to the most relevant parts of the Kikuchi patterns. Such an approach can be expected to significantly increase the sensitivity to deviations from centrosymmetry in Kikuchi electron diffraction patterns.

It is interesting to note that the (111)/($\bar{1}\bar{1}\bar{1}$) reflections and their symmetry related ones are not of the highest importance for the absolute structure determination from *X*-ray diffraction data. These reflections show high *X*-ray intensities and are significantly influenced by anomalous scattering in CoSi. Nevertheless, the large estimated standard deviation σ for the differences of the structure factors Δ*F*² reduce the influence of this Bijvoet pair on the absolute structure determination. Bijvoet pairs of much weaker reflections like ($\bar{1}14$) or ($\bar{2}24$) are more important. For example: Δ*F*^2^/*σ*: is 2.9 for (111)/($\bar{1}\bar{1}\bar{1}$): 7.7 for ($\bar{1}14$)/($1\bar{1}\bar{4}$): and 6.5 for ($\bar{2}24$)/$2\bar{2}\bar{4}$) (Supplementary Table S1).

The different influence of the absolute structure on the *X*- ray and electron backscatter diffraction patterns indicates the different dependencies of both characterization methods on structural and chemical parameters. In case of the very simple crystal structure of CoSi, the significant deviation from high symmetric position of the centrosymmetric NaCl type of structure is crucial for the detectability the non-centrosymmetry. Differences in the *X*-ray intensities of Bijvoet pairs originate from different anomalous scattering contributions of Co and Si. This chemical influence increases for reflections with high diffraction angles e.g. high Miller indexes and - consequently - the high-angle reflections dominate the Flack parameter. In contrast to *X*-ray diffraction, the formation of the Kikuchi bands originates from coherent multi-scattering of electrons and our simulation based on dynamic scattering approximation shows that the low indexed bands {111} dominate the chirality sensitivity of the EBSD patterns.

In summary, we have demonstrated that Kikuchi diffraction experiment in the scanning electron microscope provides a powerful tool to assign the absolute structure of non-centrosymmetric materials in a consistent way to *X*-ray diffraction analysis. The outline of our experiments with the independent assignment of the chirality of the same crystals allows to compare the sensitivity of both diffraction methods. Whilst X-ray diffraction is suitable for the crystal structure determination, the EBSD analysis allows the assignment of the chirality with high spatial resolution as shown on a multi-domain CoSi crystal. In this sense, EBSD represents a powerful extension to the *X*-ray diffraction method. Their combination together with state-of-the-art specimen manufacturing methods like focused-ion-beam technique opens the possibility to study the influence of non-centrosymmetry on chemical and physical properties for materials that are not accessible in form of large single crystals.

## Methods

### Single crystal preparation

Single crystals of CoSi were grown via chemical transport reaction using iodine as a transport agent^34,35^. In a first step, CoSi was synthesized by isothermal reaction of the elements cobalt (powder Alfa Aesar 99.998%) and silicon (powder Alfa Aesar 99.999%) in the presence of iodine (Alfa Aesar 99.998%) at 700 °C in evacuated fused silica tubes during 120 h. In a second step, CoSi was recrystallized during 3 weeks by a chemical transport reaction in a temperature gradient from 700 °C (source) to 800 °C (sink), and a transport agent concentration of 0.5 mg/cm^3^ iodine (Alfa Aesar 99.998%). Cross-sections of agglomerated crystals were prepared by metallographic technique (Fig. 2) and, after SiO_2_ finishing, allowed to obtain high quality EBSD patterns. Subsequently, X-ray diffraction data sets were collected using single crystal cubes (size 40 µm) which were cut out by the focused ion beam technique employing a FEI Helios G4 PFIB machine (2.5 µA, 30 kV Xe-beam).

### Electron backscatter diffraction (EBSD)

The experimental backscattered Kikuchi diffraction patterns were acquired using an EBSD system (Bruker Nano, Germany) attached to a scanning electron microscope JEOL JSM7800 F. All measured Kikuchi patterns were saved for post-processing using custom data analysis software. A best-fit pattern matching approach was used to find the orientation and the chirality corresponding to each experimental Kikuchi pattern. The normalized cross-correlation coefficient r (0 ≤ *r* ≤ 1) between the experimental and the simulated Kikuchi pattern of the reference structure was optimized according to^36^, with an additional test against the inverted structure to discriminate between the enantiomorphs. The calculation of the theoretical global reference data used for the Kikuchi pattern matching was carried out using the Bloch wave approach^32^. For each experimental pattern, two optimized correlation coefficients *r*_+E_ and *r*_−E_ were obtained from the fit to the reference structure, and the inverted enantiomorph, respectively. For the final assignment of the best-fit enantiomorph to a measured pattern and to estimate the significance of the discrimination, the difference of the correlation coefficients ∆*r* = *r*_+E_ − *r*_−E_, in relation to their average value *r*_*m*_ = (*r*_+*E*_ + *r*_−*E*_)/2 is used. A large average value *r*_m_ indicate a good fit to the simulated data, while a simultaneous large difference |∆*r*| indicates a reliable discrimination of the enantiomorphs.

### *X*-ray Diffraction

Both single-crystal specimens were investigated on a Rigaku AFC-7 diffraction system equipped with a Saturn 724 CCD detector using Mo*K*α radiation (λ = 0.71073 Å). The absorption correction of the reflection intensities was performed by multi-scan routine^37^. All crystallographic calculations were performed with the program packages WinCSD^38^ and SHELX^39^. Details of data collection and results of the structure refinement are listed in Table 1.

## Supplementary information

Absolute Structure from Scanning Electron Microscopy.

## References

[1] Barron, L. D. In Chirality at the Nanoscale (D. B. Amabilino ed.) 1 – 27 (WILEY-VCH, 2009).

[2] Flack HD (2003). Chiral and Achiral Crystal Structures. Helvetica Chimica Acta.

[3] Glazer AM, Stadnicka K (1989). On the use of the term ‘absolute’ in crystallography. Acta Crystallographica Section A.

[4] Bak P, Jensen MH (1980). Theory of helical magnetic structures and phase transitions in MnSi and FeGe. Journal of Physics C: Solid State Physics.

[5] Shibata, K. *et al*. Towards control of the size and helicity of skyrmions in helimagnetic alloys by spin–orbit coupling. *Nature Nanotechnology* **8**, 723, 10.1038/nnano.2013.174 10.1038/nnano.2013.174 24013133

[6] Fujimoto S (2007). Electron Correlation and Pairing States in Superconductors without Inversion Symmetry. Journal of the Physical Society of Japan.

[7] Bauer, E., Rogl, P. Noncentrosymmetric Superconductors: Strong vs Weak ElectronicCorrelations. (Springer, 2012).

[8] Sanchez DS (2019). Topological chiral crystals with helicoid-arc quantum states. Nature.

[9] Schröter NBM (2019). Chiral topological semimetal with multifold band crossings and long Fermi arcs. Nature Physics.

[10] Hazen RM, Sholl DS (2003). Chiral selection on inorganic crystalline surfaces. Nature Materials.

[11] Kovnir K (2007). A new approach to well-defined, stable and site-isolated catalysts. Science and Technology of Advanced Materials.

[12] Prinz J, Gröning O, Brune H, Widmer R (2015). Highly Enantioselective Adsorption of Small Prochiral Molecules on a Chiral Intermetallic Compound. Angewandte Chemie International Edition.

[13] Yakutovich AV, Hoja J, Passerone D, Tkatchenko A, Pignedoli CA (2018). Hidden Beneath the Surface: Origin of the Observed Enantioselective Adsorption on PdGa(111). Journal of the American Chemical Society.

[14] Gautier R, Klingsporn JM, Van Duyne RP, Poeppelmeier KR (2016). Optical activity from racemates. Nature Materials.

[15] Peerdeman AF, Van Bommel AJ, Bijvoet JM (1951). Determination of absolute configuration of optically active compounds by means of X-rays. Proc. K. Ned. Akad. Wet. Ser. B.

[16] Rogers, D. In Anomalous Scattering (S. Ramaseshan; S. C. Abrahams ed.) 231 – 250 (Munksgaard, 1975).

[17] Flack HD, Bernardinelli G (2008). The use of X-ray crystallography to determine absolute configuration. Chirality.

[18] Buxton BF, Eades JA, Steeds JW, Rackham GM, Frank FC (1976). The symmetry of electron diffraction zone axis patterns. Philosophical Transactions of the Royal Society of London. Series A, Mathematical and Physical Sciences.

[19] Goodman P, Secomb TW (1977). Identification of enantiomorphously related space groups by electron diffraction. Acta Crystallographica Section A.

[20] Goodman P, Johnson AWS (1977). Identification of enantiomorphically related space groups by electron diffraction - a second method. Acta Crystallographica Section A.

[21] Tanaka M, Takayoshi H, Ishida M, Endoh Y (1985). Crystal Chirality and Helicity of the Helical Spin Density Wave in MnSi. I. Convergent-Beam Electron Diffraction. Journal of the Physical Society of Japan.

[22] Inui H, Fujii A, Tanaka K, Sakamoto H, Ishizuka K (2003). New electron diffraction method to identify the chirality of enantiomorphic crystals. Acta Crystallographica Section B.

[23] Taftø J (1983). Structure-Factor Phase Information from Two-Beam Electron Diffraction. Physical Review Letters.

[24] Electron Backscatter Diffraction in Materials Science. second edition edn. (Springer, 2009).

[25] Baba-Kishi KZ, Dingley DJ (1989). Backscatter Kikuchi diffraction in the SEM for identification of crystallographic point groups. Scanning.

[26] Winkelmann A, Nolze G (2015). Point-group sensitive orientation mapping of non-centrosymmetric crystals. Applied Physics Letters.

[27] Winkelmann A, Nolze G (2015). Chirality determination of quartz crystals using Electron Backscatter Diffraction. Ultramicroscopy.

[28] Burch MJ, Fancher CM, Patala S, De Graef M, Dickey EC (2017). Mapping 180° polar domains using electron backscatter diffraction and dynamical scattering simulations. Ultramicroscopy.

[29] Nolze G, Grosse C, Winkelmann A (2015). Kikuchi pattern analysis of noncentrosymmetric crystals. Journal of Applied Crystallography.

[30] Koch, E., Fischer, W. In International Tables for Crystallography Vol. A (ed. Hahn, T.) Ch. 15, 877–905 (Kluwer Academic Press, 2002).

[31] Spence JCH, Zuo JM, O’Keeffe M, Marthinsen K, Hoier R (1994). On the minimum number of beams needed to distinguish enantiomorphs in X-ray and electron diffraction. Acta Crystallographica Section A.

[32] Winkelmann A, Trager-Cowan C, Sweeney F, Day AP, Parbrook P (2007). Many-beam dynamical simulation of electron backscatter diffraction patterns. Ultramicroscopy.

[33] Winkelmann A (2008). Dynamical effects of anisotropic inelastic scattering in electron backscatter diffraction. Ultramicroscopy.

[34] Bosholm, O., Oppermann, H. & Däbritz, S. In *Zeitschrift für Naturforschung B* Vol. 55, 1199 (2000).

[35] M., Binnewies, R. G., M. Schmidt, P. Schmidt. Chemical Vapor Transport Reactions. (de Gruyter, 2013).

[36] Nolze G, Hielscher R, Winkelmann A (2017). Electron backscatter diffraction beyond the mainstream. Crystal Research and Technology.

[37] Blessing RH (1995). An empirical correction for absorption anisotropy. Acta Crystallographica Section A.

[38] Akselrud L, Grin Y (2014). WinCSD: software package for crystallographic calculations (Version 4). Journal of Applied Crystallography.

[39] Sheldrick G (2015). Crystal structure refinement with SHELXL. Acta Crystallographica Section C.

