# Supplementary material for: Absolute Structure from Scanning Electron Microscopy
Source: Sci Rep. 2020 Mar 4;10:4065. doi: 10.1038/s41598-020-59854-y (PMC7055257; doi:10.1038/s41598-020-59854-y)

Supplementary

**Absolute Structure from Scanning Electron Microscopy**

Ulrich Burkhardt, Horst Borrmann, Philip Moll, Marcus Schmidt, Yuri Grin,

Aimo Winkelmann


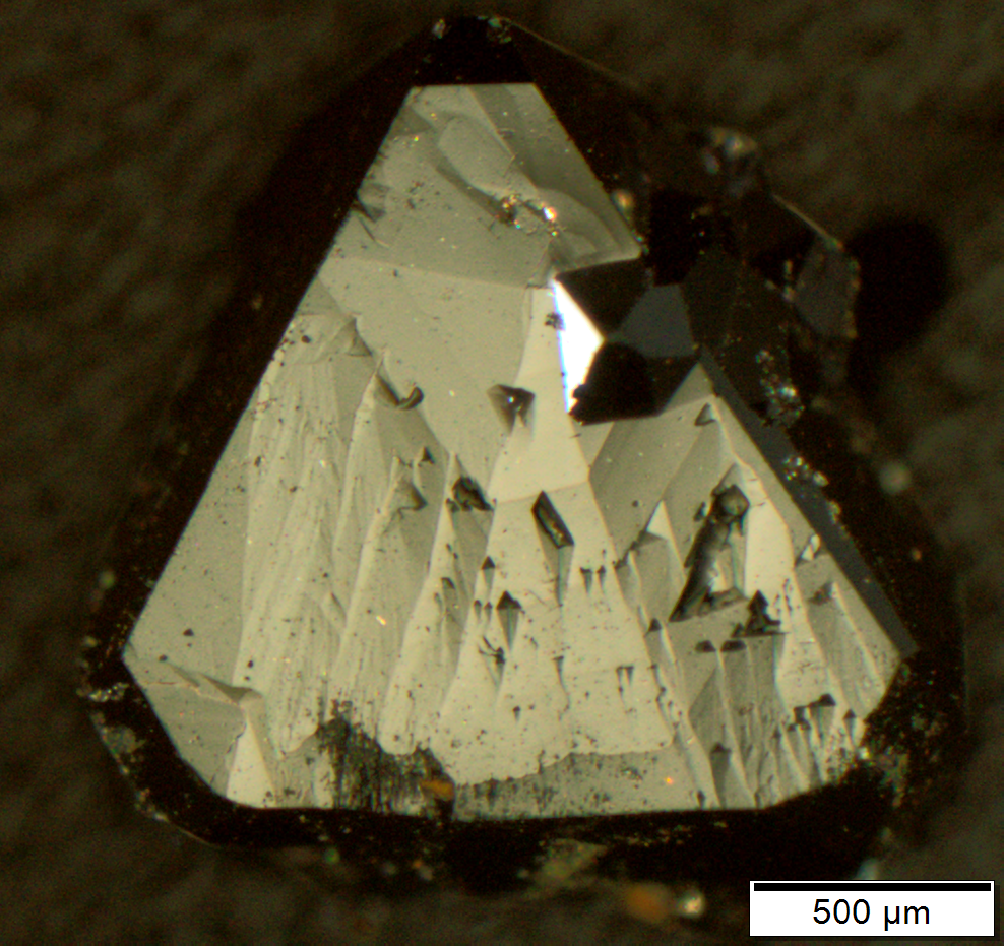


Figure S1: Surface of as grown CoSi crystal with small crystal on top (light optical image, bright field contrast).

Table S1: Comparison of *F*² values of Bijvoet – pairs (*hkl*)_1_/(*hkl*)_2_ derivated from *X*-ray diffraction data of CoSi crystals 1 and 2. The ratio $\frac{\boldsymbol{\Delta}\boldsymbol{F}^{\boldsymbol{2}}}{\boldsymbol{\sigma}}$ (σ …esd of Δ*F*^2^) indicate on the statistical relevance for the determination of the absolute structure.

|  |  | **Crystal 1** | | | |  | **Crystal 2** | | | |
| --- | --- | --- | --- | --- | --- | --- | --- | --- | --- | --- |
| **(h k l)_1_** | **(h k l)_2_** | $\boldsymbol{F}_{\boldsymbol{(}\boldsymbol{hkl)}_{\boldsymbol{1}}}^{\boldsymbol{2}}$ | $\boldsymbol{F}_{\boldsymbol{(}\boldsymbol{hkl)}_{\boldsymbol{2}}}^{\boldsymbol{2}}$ | **ΔF^2^**  **(**$\boldsymbol{F}_{\boldsymbol{(}\boldsymbol{hkl)}_{\boldsymbol{1}}}^{\boldsymbol{2}}$**-** $\boldsymbol{F}_{\boldsymbol{(}\boldsymbol{hkl)}_{\boldsymbol{2}}}^{\boldsymbol{2}}$**)** | $\frac{\boldsymbol{\Delta}}{\boldsymbol{\sigma}}$ |  | $\boldsymbol{F}_{\boldsymbol{(}\boldsymbol{hkl)}_{\boldsymbol{1}}}^{\boldsymbol{2}}$ | $\boldsymbol{F}_{\boldsymbol{(}\boldsymbol{hkl)}_{\boldsymbol{2}}}^{\boldsymbol{2}}$ | **ΔF^2^**  **(**$\boldsymbol{F}_{\boldsymbol{(}\boldsymbol{hkl)}_{\boldsymbol{1}}}^{\boldsymbol{2}}$**-** $\boldsymbol{F}_{\boldsymbol{(}\boldsymbol{hkl)}_{\boldsymbol{2}}}^{\boldsymbol{2}}$**)** | $\frac{\boldsymbol{\Delta}}{\boldsymbol{\sigma}}$ |
| $\overline{1}14$ | $114$ | 556 | 487 | 70 | 7.7 |  | 486 | 552 | -66 | 9.6 |
| $\overline{2}24$ | $224$ | 52 | 33 | 19 | 6.5 |  | 32 | 51 | -19 | 8.2 |
| $\overline{1}24$ | $124$ | 312 | 361 | -50 | 6.3 |  | 356 | 321 | 34 | 6.3 |
| $\overline{2}23$ | $223$ | 37 | 51 | -13 | 6.3 |  | 50 | 36 | 14 | 7.6 |
| $\overline{2}13$ | $213$ | 285 | 256 | 29 | 6.1 |  | 256 | 291 | -35 | 7.2 |
| $\overline{1}36$ | $136$ | 60 | 34 | 25 | 5.5 |  | 35 | 58 | -23 | 6.1 |
| $\overline{1}22$ | $122$ | 441 | 407 | 33 | 4.8 |  | 398 | 433 | -35 | 5.0 |
| $\overline{1}16$ | $116$ | 204 | 243 | -39 | 4.5 |  | 243 | 201 | 42 | 5.1 |
| $\overline{1}26$ | $126$ | 68 | 51 | 17 | 4.3 |  | 50 | 67 | -17 | 4.5 |
| $\overline{1}44$ | $114$ | 370 | 333 | 37 | 4.0 |  | 326 | 364 | -38 | 5.0 |
| $\overline{2}44$ | $244$ | 79 | 101 | -22 | 4.0 |  | 99 | 74 | 25 | 5.3 |
| $\overline{1}34$ | $134$ | 216 | 252 | -36 | 4.0 |  | 246 | 217 | 29 | 4.9 |
| $\overline{3}44$ | $344$ | 49 | 71 | -22 | 3.9 |  | 73 | 48 | 25 | 4.6 |
| $\overline{1}13$ | $113$ | 983 | 1047 | -65 | 3.9 |  | 103 | 991 | 39 | 4.6 |
| $\overline{2}34$ | 234 | 269 | 243 | 26 | 2.9 |  | 251 | 270 | -19 | 3.7 |
| $\overline{3}14$ | $314$ | 643 | 691 | -49 | 2.9 |  | 699 | 644 | 56 | 3.9 |
| $\overline{1}11$ | $111$ | 1222 | 1007 | 215 | 2.9 |  | 1037 | 1111 | -74 | 4.4 |
| $\overline{3}46$ | $346$ | 65 | 49 | 16 | 2.9 |  | 45 | 65 | -20 | 3.8 |
| $\overline{4}16$ | $416$ | 237 | 268 | -31 | 2.7 |  | 273 | 230 | 42 | 3.4 |
| $\overline{3}26$ | $326$ | 28 | 39 | -11 | 2.6 |  | 39 | 27 | 12 | 3.1 |
| $\overline{2}17$ | $217$ | 12 | 19 | -6 | 2.5 |  | 19 | 12 | 7 | 2.9 |
| $\overline{3}25$ | $325$ | 7 | 3 | 4 | 2.5 |  | 3 | 7 | -4 | 3.2 |
| $\overline{1}46$ | $146$ | 248 | 289 | -41 | 2.3 |  | 291 | 254 | 37 | 2.9 |
| $\overline{2}46$ | $246$ | 74 | 63 | 11 | 2.3 |  | 60 | 74 | -13 | 2.7 |
| $\overline{4}26$ | $426$ | 79 | 66 | 13 | 2.2 |  | 64 | 78 | -14 | 2.6 |
| $\overline{1}17$ | $117$ | 202 | 184 | 18 | 2.1 |  | 184 | 196 | -12 | 2.2 |
| $\overline{2}27$ | $227$ | 13 | 8 | 5 | 2.1 |  | 9 | 13 | -5 | 2.3 |
| $\overline{3}34$ | $334$ | 174 | 158 | 15 | 1.9 |  | 157 | 173 | -16 | 2.3 |

**Estimation of the significance of cross correlation differences for the assignment of chirality from EBSD patterns**

The assignment of chirality from EBSD patterns by the pattern matching method is based the quantitative comparison of measured and simulated EBSD patterns, using the normalized cross correlation coefficient as the image similarity metric. The EBSD patterns of both enantiomorphs are very similar and lead to closely similar values of the cross correlation coefficients *r*_+E_ and *r*_-E_ which measure the agreement of the experimental and the simulated patterns of both enantiomorphs. The geometric mean (*r*_+E_ + *r*_-E_)/2 gives the overall agreement between measured and simulated data and is dominated by experimental uncertainties if the structure model is correct. The value (∆*r* = *r*_+E_ - *r*_-E_) indicates which of the two possible simulated enantiomorph patterns is in better agreement with the measured pattern. Therefore, it is necessary to discuss which changes in ∆*r* lead to a statistically significant identification of an enantiomorph, as compared to changes in ∆*r* which are purely due to the experimental noise.

In order to estimate significant values of ∆*r*, we use a comparison of the ideal enantiomorph patterns with data which was simulated according to a phenomenological detection model of EBSD. Specifically, we test the null hypothesis that the observed values of ∆*r* ≠ 0 are purely due to statistical variations of a detected pattern which originates from 50/50 mixture of both enantiomorphs (for which ideally we should have ∆*r* = 0). The null hypothesis basically states that the experimental Kikuchi pattern does ***not*** contain information about the chirality of the crystal.

In order to obtain an estimation of the probability distribution of ∆*r* under the null hypothesis, we have carried out a simulation of 10000 pseudo-experimental patterns which include the phenomenological effects due to electron counting and camera noise, a spatially varying background intensity, excess-deficiency effects of the Kikuchi bands and lens-blurring of the optical system of the EBSD detector. These parameters can be adjusted to obtain simulated pseudo-experimental patterns which account for different noise levels in real experimental patterns. Using the 10000 pseudo-experimental patterns, we carried out exactly the same image processing and pattern matching process as in the experiment.

In Figure S2, we show an example comparison for a mean *r*_m_ near 0.42, with a difference of ∆*r* = -0.002 apparently indicating a better fit of model B in this specific realization out of the 10000 samples. We note that this difference is produced purely by statistical variations, as both theories A and B fit equally well to the average centrosymmetric pattern that underlies the detection model.


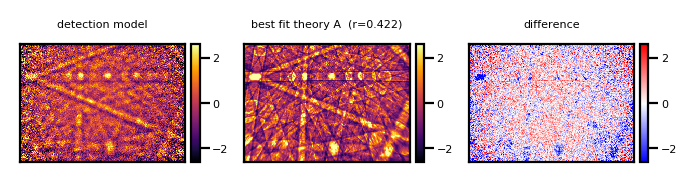
_
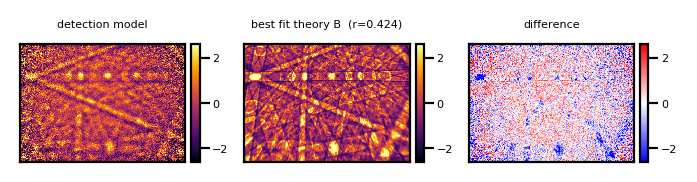
_

Figure S2: Simulated experimental pattern matching using a model for the physical EBSD measurement process. The theoretical pattern which is the basis for the pseudo-experimental pattern shown as “detection model” does ***not*** contain any non-centrosymmetric crystallographic effects and any apparently better fit of model A vs. B is due to statistical variations affecting the fit process.

The histogram of the simulated distribution of ∆*r* is shown in Figure S3. Assuming that the histogram can be described approximately by a Gaussian distribution centered around a zero mean, with standard deviation σ, we find a value of 3σ = 0.0072. This means that there is a probability of less than 0.3% to find differences with |∆*r*| > 0.01 for the considered case where the noise and experimental effects produce an observed mean _‘_cross correlation value of *r*_m_ = 0.42. Based on the significance level of 0.3%, we thus can *reject* the null hypothesis, i.e. changes of ∆*r* > 0.01 are statistically significant for a mean *r*_m_ near 0.4 and we can discriminate enantiomorphs based on this estimation. The estimation of ∆*r* > 0.01 will be valid also for *r*_m_ > 0.4, as even smaller ∆*r* will tend to be relevant at higher *r*_m_.


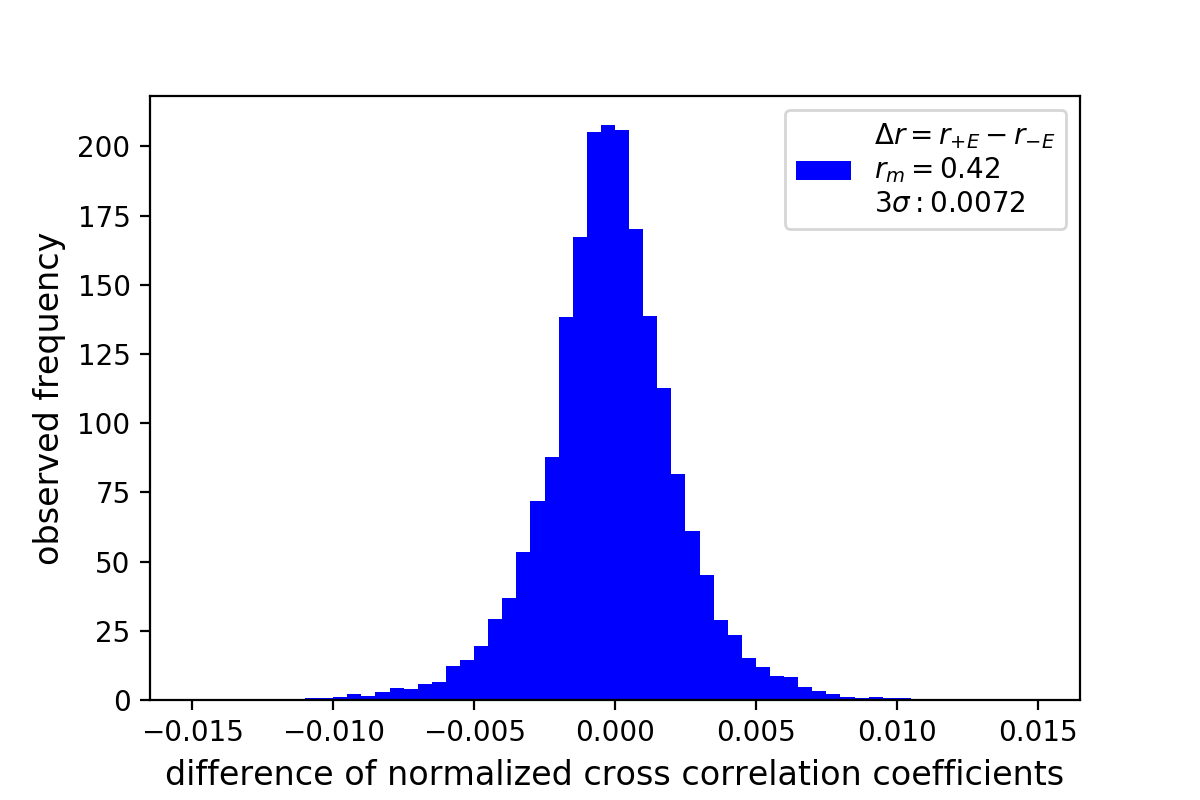
In case of our experimental EBSD measurements on the non-centrosymmetric phase CoSi, the cross-correlation coefficient between the experimental and simulated patterns shows values of *r*_+E_ and *r*_-E_ close to 0.6 (*r*_m_ = 0.6) and the differences reach values |∆*r*| = 0.02. This difference is thus significantly large because, as estimated above, it cannot be expected to be caused by experimental noise and a detection of a pattern from a hypothetical centrosymmetric structure or from comparison with a pattern from an area with twinned domains (based on a significance level <0.3%).

Figure S3: Distribution of the difference value (∆*r* = *r*_+E_ - *r*_-E)_) between both cross-correlation coefficients *r*_+E_, *r*_-E_ measuring the similarity between simulated pattern of both enantiomorphs and the pseudo-experimental centrosymmetric reference patterns as explained in the text.


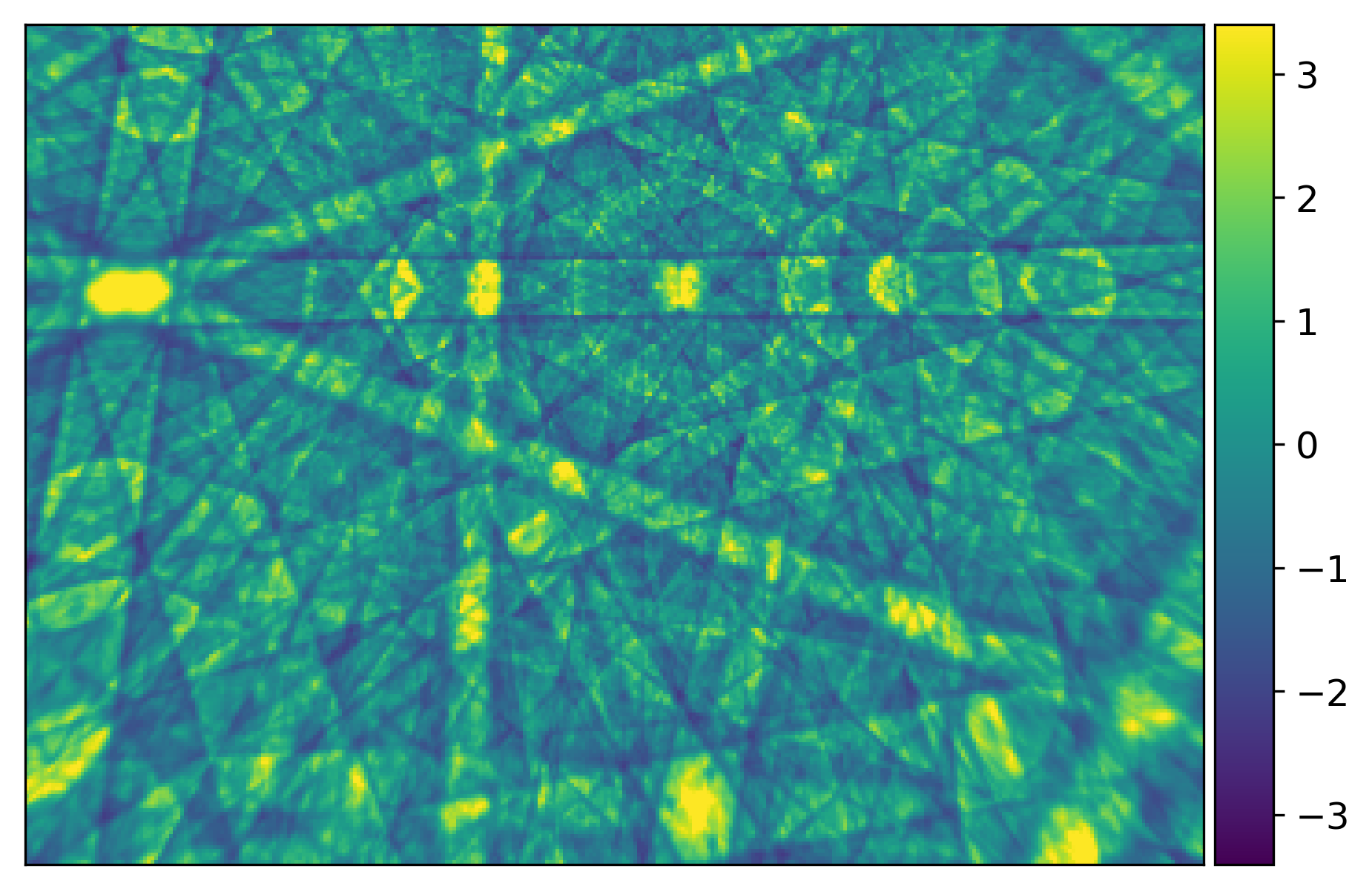

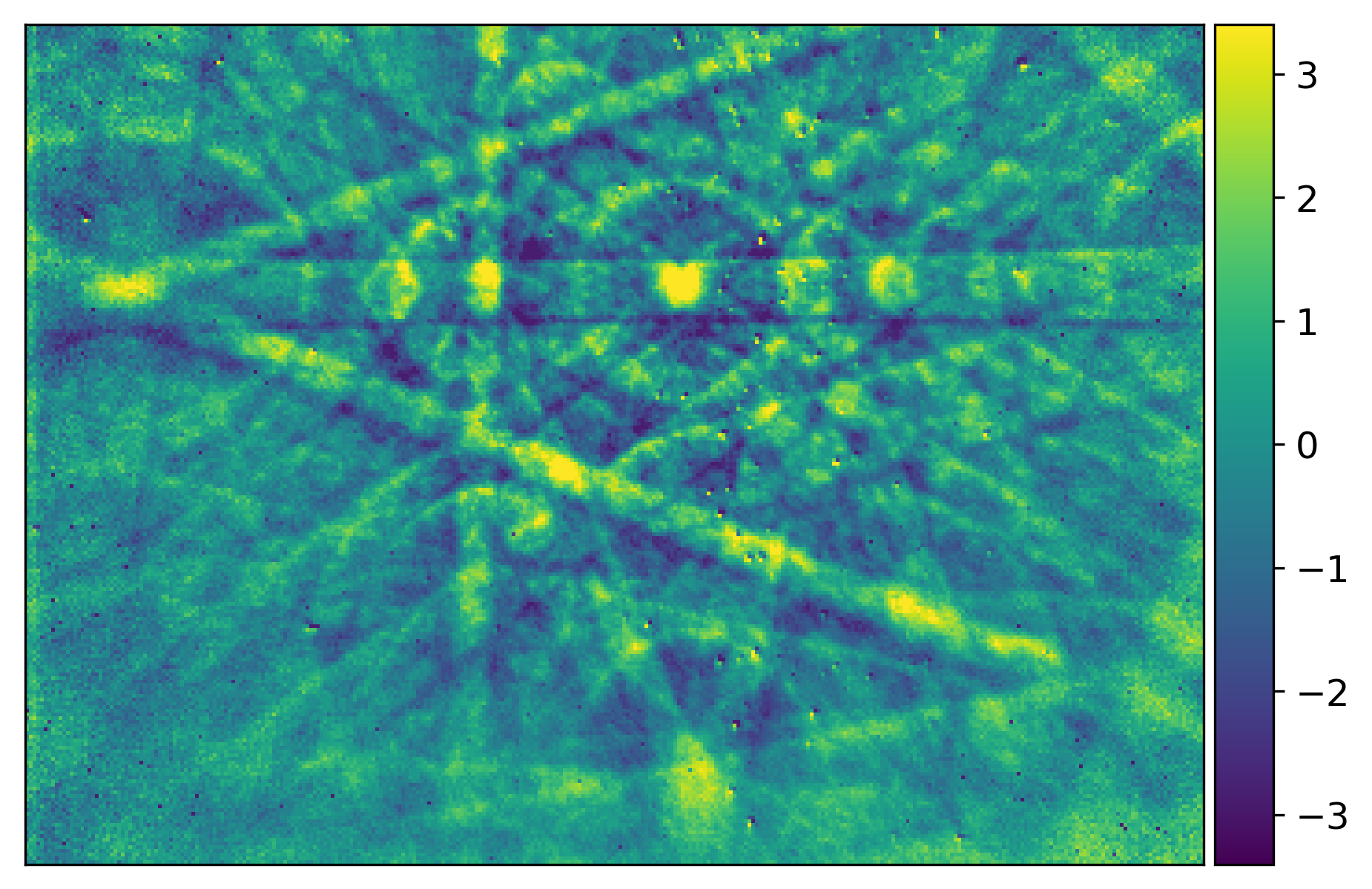
Figure S4-1:

Top: **’M’** region; measured EBSD pattern

bottom**: A form**; simulated EBSD pattern

*visualizing chirality-dependent differences*

(use page by page presentation; turn page forward →)


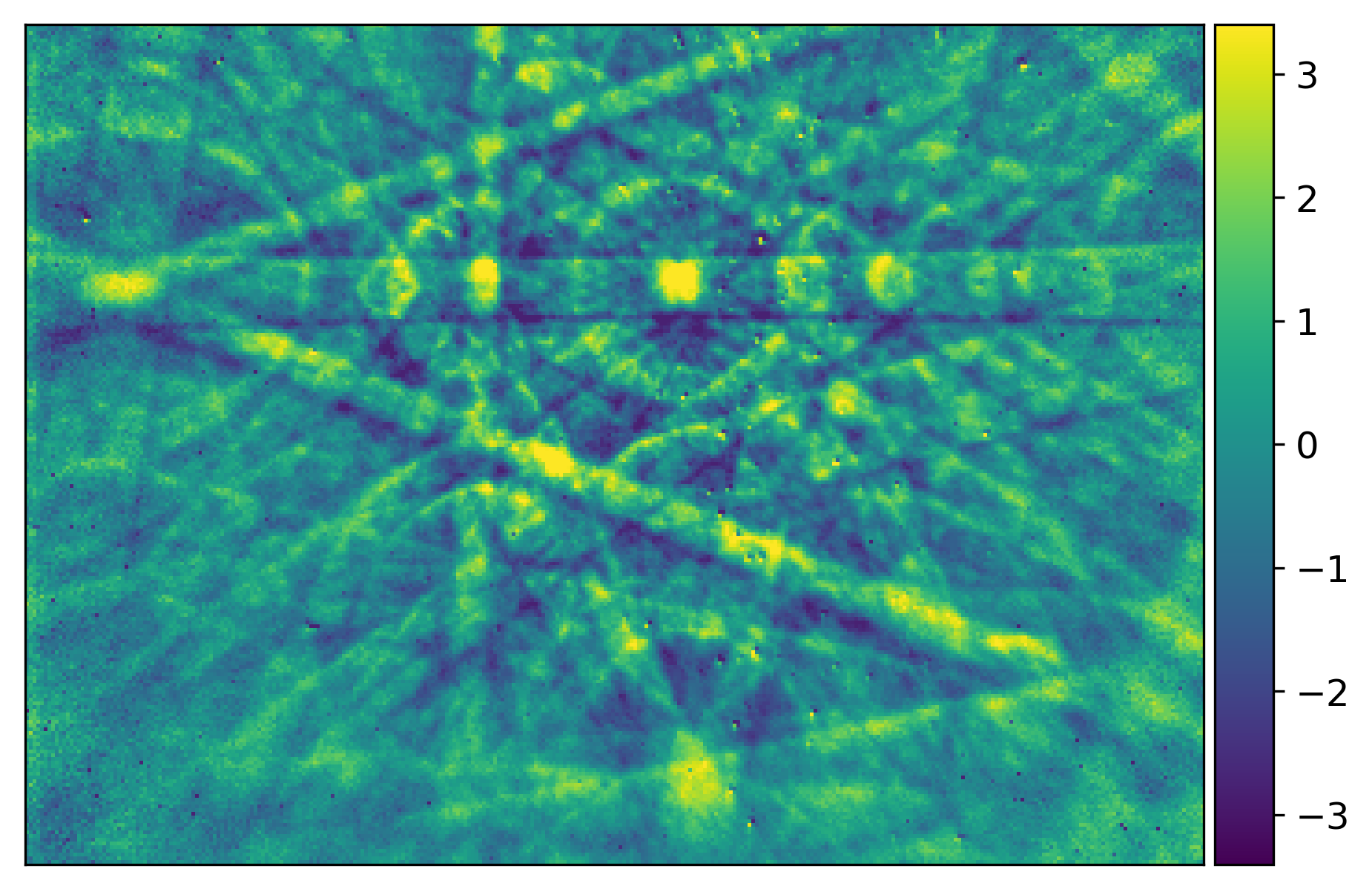
Figure S4-2:

Top :**’T’** region; measured EBSD pattern

bottom: **B form**: simulated EBSD pattern

*visualizing chirality-dependent differences*

(use page by page presentation; turn page backward ←)
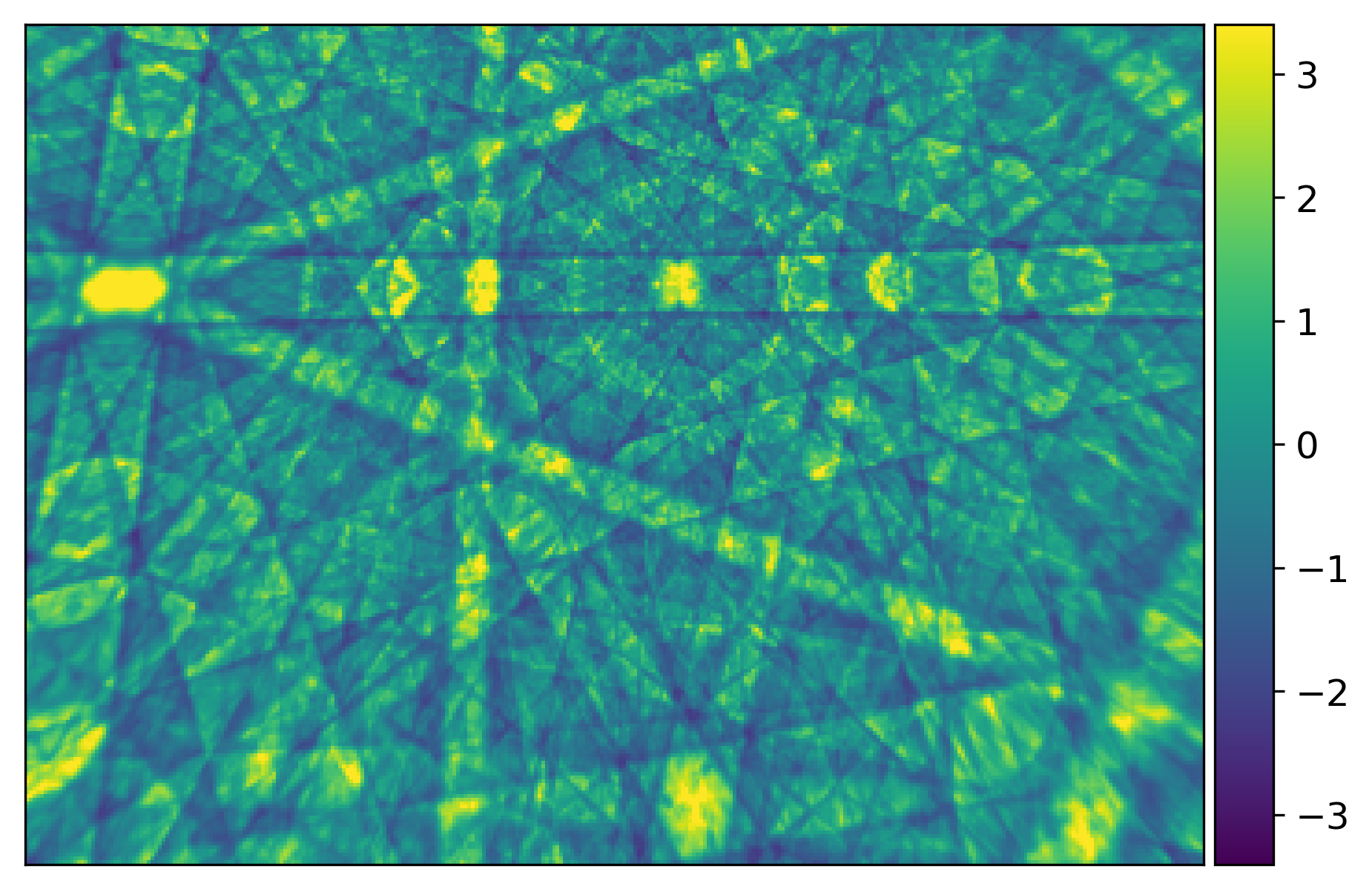

Supplement: Supplementary file 1 — Absolute Structure from Scanning Electron Microscopy. [file 41598_2020_59854_MOESM1_ESM.docx]
